# Life cycle of *Renylaima capensis*, a brachylaimid trematode of shrews and slugs in South Africa: two-host and three-host transmission modalities suggested by epizootiology and DNA sequencing

**DOI:** 10.1186/1756-3305-5-169

**Published:** 2012-08-13

**Authors:** Wilhelm F Sirgel, Patricio Artigas, M Dolores Bargues, Santiago Mas-Coma

**Affiliations:** 1 Department of Botany and Zoology, University of Stellenbosch, Private Bag X1, Matieland, 7602, South Africa; 2 Departamento de Parasitología, Facultad de Farmacia, Universidad de Valencia, Av. Vicente Andrés Estellés s/n, 46100, Burjassot - Valencia, Spain

## Abstract

**Background:**

The life cycle of the brachylaimid trematode species *Renylaima capensis*, infecting the urinary system of the shrew *Myosorex varius* (Mammalia: Soricidae: Crocidosoricinae) in the Hottentots Holland Nature Reserve, South Africa, has been elucidated by a study of its larval stages, epizootiological data in local snails and mammals during a 34-year period, and its verification with mtDNA sequencing.

**Methods:**

Parasites obtained from dissected animals were mounted in microscope slides for the parasitological study and measured according to standardized methods. The mitochondrial DNA *cox*1 gene was sequenced by the dideoxy chain-termination method.

**Results:**

The slugs *Ariostralis nebulosa* and *Ariopelta capensis* (Gastropoda: Arionidae) act as specific first and second intermediate hosts, respectively. Branched sporocysts massively develop in *A. nebulosa*. Intrasporocystic mature cercariae show differentiated gonads, male terminal duct, ventral genital pore, and usually no tail, opposite to Brachylaimidae in which mature cercariae show a germinal primordium and small tail. Unencysted metacercariae, usually brevicaudate, infect the kidney of *A. capensis* and differ from mature cercariae by only a slightly greater size. The final microhabitats are the kidneys and ureters of the shrews, kidney pelvis and calyces in light infections and also kidney medulla and cortex in heavy infections. Sporocysts, cercariae, metacercariae and adults proved to belong to *R. capensis* by analysis of a 437-bp-long *cox*1 fragment, which was identical except for three mutations in metacercariae, of which only one silent. Epizootiological studies showed usual sporocyst infection in *A. nebulosa* and very rare metacercarial infection in *A. capensis*, which does not agree with high prevalences and intensities in the shrews.

**Conclusions:**

The presence of monotesticular adult forms and larval prevalences and intensities observed suggest that *R. capensis* may use two transmission strategies, a two-host life cycle by predation of *A. nebulosa* harbouring intrasporocystic cercariae may be the normal pattern, whereas a second mollusc host is just starting to be introduced. In shrews, a tissue-traversing, intraorganic migration followed by an interorganic migration to reach and penetrate the outer surface of either of both kidneys should occur. For first slug infection, the fluke takes advantage of the phenomenon that *M. varius* always urinate during defaecation. Consequently, in Brachylaimidae, the second intermediate mollusc host should evolutionarily be seen as a last addition to the cycle and their present adult stage microhabitat restricted to digestive tract and related organs as a loss of the tissue-traversing capacity of the metacercaria.

## Background

Brachylaimidae are an interesting family of trematodes due to their numerous species as well as their worldwide presence and frequency. Brachylaimids are also of medical importance as they cause diseases in humans, which can even result in mortality [1,2]. In the veterinary field, they affect domestic animals [3], poultry [4-8] and wild game birds [9,10]. They have also proved to be useful as biological tags in studies of postfire ecosystem regeneration processes [11,12]. Additionally, Brachylaimidae are biologically peculiar. While the great majority of digenean trematodes follow an aquatic life cycle [13], brachylaimids are one of the very few trematode groups (the only other one is Dicrocoeliidae) that have succeeded in colonizing the terrestrial milieu, in some instances even very arid, xerophilic habitats [14].

Brachylaimid species of the three subfamilies Brachylaminae, Ityogoniminae and Panopistinae [15] share the same three-host life cycle pattern including (i) a terrestrial gastropod snail (Pulmonata: Stylommatophora) as first intermediate host, (ii) an additional mollusc individual belonging to another (or sometimes the same) terrestrial gastropod species as second intermediate host, and (iii) an endothermic vertebrate as definitive host [13,14,16-18].

The first snail host is very specific, as brachylaimid species use only one or a very small number of closely related mollusc species inhabiting the local area [14,16,17]. This mollusc becomes infected by eating embryonated fluke eggs shed in faeces by the definitive host. The first sporocystogenous sporocyst generation develops in the hepatopancreas [19-22]. This early mother sporocyst phase constitutes the only plathyhelminth stage known to develop inside a host cell [23]. There are no rediae in brachylaimids, so that cercariae are directly produced inside second sporocyst generations. These cercariogenous sporocysts develop primarily in the hepatopancreas, although other secondary locations become invaded by these sporocysts in massive infections [24], and show a very characteristic branched morphology [25]. Cercariae are brevicaudate (similar to the microcercous type of aquatic cercariae but lacking a stylet) and are shed by the first intermediate snail host under rainy conditions or high humidity, so that either water from rain or snail individuals cohabiting under close contact (as under big stones) ensure the survival of the free and shortliving cercaria thus enabling transmission to a second intermediate snail host [26-28].

The second snail host appears to be less specific. It usually belongs to another terrestrial snail species sharing the same habitat with the first one. Sometimes it may even be other individuals of the first host species. Cercariae penetrate through orifices of the body and migrate to the intramolluscan microhabitat where they will develop into an unencysted metacercarial stage. The microhabitat of metacercariae appears to be very specific [14,16,17]. The location of metacercariae is the kidney for most of the brachylaimid species, although pericardial cavity and rarely pedal glands are microhabitats for a few species.

Definitive hosts of brachylaimids are homeothermic mammals and birds normally feeding on snails or accidentally ingesting them with other food (i.e., in pigs) [15]. Many brachylaimid species show a clearly restricted spectrum of closely related definitive host species (or sometimes even a single one), suggesting a phylogenetic specificity, as is the case of species infecting mammals of the Suborder Soricomorpha (shrews Soricidae and moles Talpidae). Within mammals, insectivores, rodents and marsupials are the main definitive hosts. Artiodactyls (pigs Suidae), carnivores and lagomorphs are only secondarily captured hosts.

In Brachylaimidae, definitive hosts other than mammals and birds are very rare. Another subfamily Zeylanurotrematinae, suggestedly close to Panopistinae, has been proposed within Brachylaimidae to include flukes infecting reptiles and amphibians [29]. Even family rank (Zeylanurotrematidae) has recently been proposed for them [30], although the systematic position of these flukes has remained *incertae sedis* in the most recent review of the trematode group [31]. Another rare, monotesticular species, *Parabrachylaima euglandensis*, whose adult stage progenetically develops in terrestrial snails of Lousiana, USA [32], has also recently been retained within Brachylaimidae [33].

The adult stage of brachylaimids is intestinal, including species which are haematophagous [16]. Only a very few species present an adult stage infecting other parts of the digestive tract or organs directly related or connected to the digestive tract: *Brachylaima oesophagei* and *B. fulvus* in the oesophagus and stomach [34,35], *Scaphiostomum* species in ducts of liver and pancreas [36], and *Dollfusinus frontalis* in nasal and frontal sinuses [14,37]. Exceptions are the lumen of the kidney sac as final microhabitat for *Parabrachylaima*[32], and the urinary system for species of *Zeylanurotrema*[29,38], although a species of the latter, *Z. sphenomorphi*, has been described from the host’s intestine [30].

Recently, a new curious brachylaimid species was described based on adult trematode specimens found in the kidneys and ureters of the forest shrew *Myosorex varius* (Smuts, 1832) (Insectivora: Soricidae: Crocidosoricinae) from a restricted, very damp area of the Hottentots Holland Mountain range, near Cape town, South Africa. Peculiar characteristics such as the absence of a cirrus pouch and cirrus, the presence of a genital atrium that can be evaginated to produce a prominent ventral extention of the body, and a surprising 11.1% of monotesticular forms due to the lack of an anterior testis, as well as the fact that the urinary system of a mammal host is an extraordinary microhabitat for a trematode, indicated that this digenean represents a new genus and species for which the name *Renylaima capensis* was proposed [39]. The present article aims to describe the elucidation of its life cycle and transmission by means of the parasitological study of its larval stages, its epizootiological data in local snail and shrew hosts, and its verification with mtDNA marker sequencing.

## Methods

### Host materials

**Figure 1:**
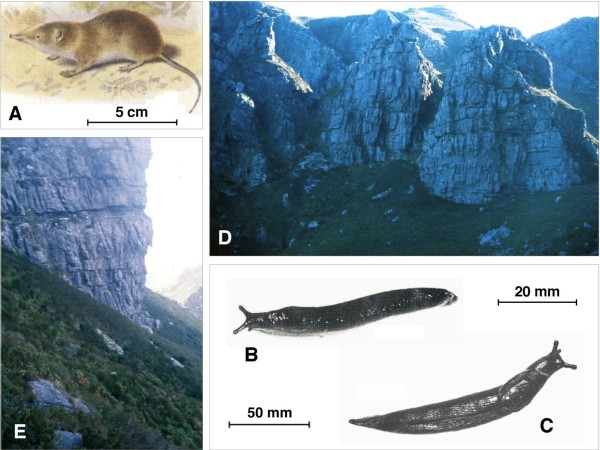
**Environment and hosts of *Renylaima capensis* .A**) drawing showing the forest shrew *Myosorex varius* (Insectivora: Soricidae) (from [70]); **B**) slug specimen of the species *Ariostralis nebulosa* (Gastropoda: Arionidae: Ariopeltinae); **C**) slug specimen of the species *Ariopelta capensis* (Gastropoda: Arionidae: Ariopeltinae); **DE**) general views of the area near the top end of a narrow ravine called Landdroskloof, on the western slopes of the Hottentots Holland Mountains, where the life cycle of the new brachylaimid develops; this area is situated at the foot of a high southward facing cliff which shades off the sun for the major part of each day (1350 m altitude); south facing cliffs bordering and partly enclosing the habitat on its northern side (**D**) are approximally 35 m high. Scale bars: **A** = 5 cm; **B** = 20 mm; **C** = 50 mm.

Since the year 1978, shrews have been trapped (Figure 1A) and land snails hand collected (Figure 1B, C) from a moist habitat (34° 02′ 48.5″ S; 18° 59′ 11.6″ E) of the Hottentots Holland Nature Reserve, about 60 km to the east of Cape town and thus in the most southern part of South Africa in what is now called the Western Province of the Republic of South Africa. Trematode adults and larval stages were collected after host dissection under a stereomicroscope.

All the infected slugs and shrews were collected in an area limited to about 1000 m^2^ near the top end of a narrow ravine called Landdroskloof, on the western slopes of the Hottentots Holland Mountains. The locality is at an altitude of 1350 m above sea level and situated at the foot of a high southward facing cliff which shades off the sun for the major part of each day. This area is normally covered by mist accompanied by rain or drizzle for at least a few hours of most days of the year, ensuring cool and damp conditions. The vegetation consists of a dense growth of Cyperaceae, Restionaceae, fungi and moss of the genus *Lophocolea* (Figure 1D, E).

### **Ethical statement**

Both definitive shrew hosts and intermediate snail hosts were collected in a protected area of the Hottentots Holland Nature Reserve after obtaining of the appropriate official permissions from Cape Nature to collect in the area under their jurisdiction. Restriction rules on limited specimen number collection per year were followed. For this reason, a very long period of 34 years was required until sufficient numbers of host specimens had been collected, so that significant conclusions could be made. Due to the restriction reasons, no laboratory adaptation of the hosts involved could be assayed and an indirect strategy by means of epizootiological data analysis and DNA marker sequencing of the different adult and larval stages has been used to elucidate the life cycle and transmission modalities of the brachylaimid in question. Animal ethics guidelines were strictly adhered to in the management of the animals.

### Parasitological techniques

Larval stages were obtained from snail hosts. They were initially shortly studied alive in a thin layer of water in a Petri dish under the microscope, and immediately fixed in alcohol 70%. Parts of the extensively ramified sporocysts were dissected out from snail tissues. A whole general description was made from non-mounted snail parts obtained after dissection and a detailed higher resolution analysis of terminal and subterminal sporocyst branches could be performed from fragments cut, stained with Grenacher’s borax carmine and mounted in Canada balsam to be studied in microscopic slide preparations. Terminology and measurements of sporocysts follow the standards previously proposed for brachylaimids [25].

Fully developed, mature cercariae, obtained by dissection of terminal sporocyst branches, as well as metacercariae, were also stained with Grenacher’s borax carmine and mounted in Canada balsam to be studied as whole mounts.

Adult stages obtained from shrew kidneys and ureters were fixed in Bouin’s fluid between slide and cover glass to obtain flattened specimens, and afterwards stained with Grenacher’s borax carmine and mounted in Canada balsam for microscope studies as whole mounts. Other adult specimens were fixed in alcohol 70% for DNA extraction and sequencing purposes.

Measurementes were made following the standardized method proposed for brachylaimid trematodes [40] and are given in micrometres as ranges with the mean in parentheses. Drawings were made with the aid of a camera lucida.

### DNA marker sequencing

A fragment of the cytochrome c oxidase subunit I (*cox*1) coding gene of the mitochondrial DNA was used as a barcode. This mitochondrial gene was selected according to its genetic characteristics of relatively fast evolution and verified intraspecific marker usefulness in invertebrates [41,42]. DNA was extracted from the alcohol-fixed sporocysts, cercariae, metacercariae from snails and adults from shrews. Total DNA was isolated according to the phenol-chloroform extraction and ethanol precipitation method. The procedure steps were performed according to methods outlined previously [43]. The pellet was dried and resuspended in 30 μl sterile TE buffer (pH 8.0). This suspension was stored at –20°C until use.

The mitochondrial DNA marker selected was PCR amplified independently for each specimen and each PCR product was sequenced for a bona-fide haplotype characterisation. The *cox*1 gene fragment was amplified using specific primers already used in trematodes [44,45]. Amplifications were generated in a Mastercycle ep*gradient* (Eppendorf, Hamburg, Germany), using specific PCR conditions as previously described [44,45]. Ten μl of each PCR product were checked by staining with ethidium bromide on 1% Nusieve® GTG agarose (FMC) gel electrophoresis, using the Molecular Weight Marker VI (Boehringer Mannheim) at 0.1 μg DNA/μl as control.

Primers and nucleotides were removed from PCR products by purification on Wizard™ PCR Preps DNA Purification System (Promega, Madison, WI, USA) according to the manufacturer’s protocol and suspended in 50 μl of 10 mM TE buffer (pH 7.6). The final DNA concentration was determined by measuring the absorbance at 260 and 280 nm.

DNA sequencing was performed on both strands by the dideoxy chain-termination method [46]. It was carried out with the Taq dye-terminator chemistry kit for ABI 3730 DNA Analyzer (Applied Biosystems, Foster City, CA, USA), using PCR primers. Sequences were compared by alignment using CLUSTAL-W version 1.8 [47]. Homologies were analysed using the BLASTN programme from the National Center for Biotechnology information website (http://www.ncbi.nlm.nih.gov/BLAST).

The codes for the sequences obtained follow the standard nomenclature previously proposed for other trematodes and snails [48,49]. It shall be noted that haplotype codes are only definitive in the case of complete sequences. When dealing with fragments or incomplete sequences, haplotype codes are provisional.

Sequence data of *cox*1 nucleotides and COX1 amino acids reported in this article are available in the EMBL database under the accession numbers noted below.

## Results

### Intermediate snail hosts

Only five species of pulmonates occur in the small area where the fluke species was found. Three are small snail species of *Trachycystis* (Endodontidae), a genus widely spread throughout South Africa with numerous species: *T. leucocarina, T. contrasta* and *T. cosmia*. The remaining two are the slugs *Ariostralis nebulosa* and *Ariopelta capensis* (Arionidae: Ariopeltinae) (Figure 1B, C) [50]. Both slug species have been observed to be eaten by the shrew *Myosorex varius*.

**Figure 2:**
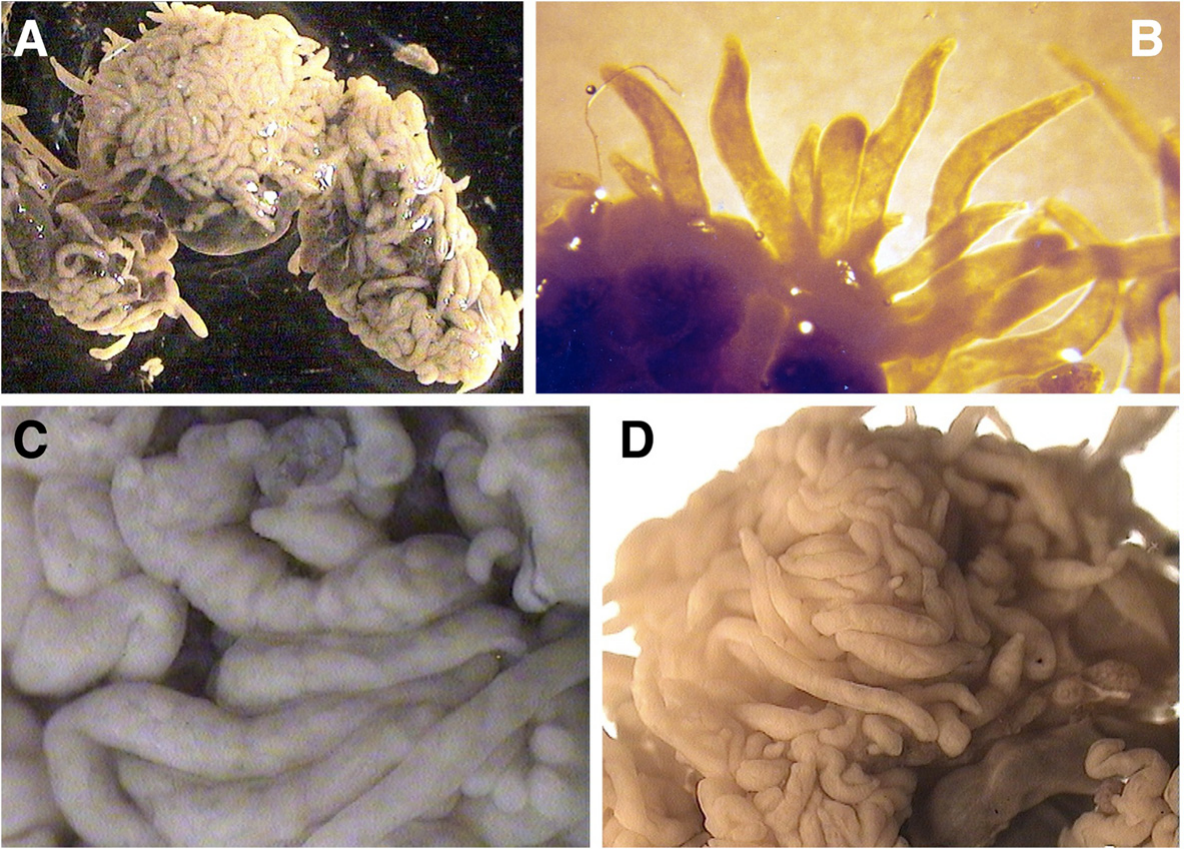
**Sporocysts of *Renylaima capensis* in the slug species *Ariostralis nebulosa.* A**) massive infection in the first intermediate host; **B**) ramified sporocyst showing terminal branches; **C**, **D**) dilated long branches including very numerous cercariae (note absence of secondary budding in the branches).

**Figure 3:**
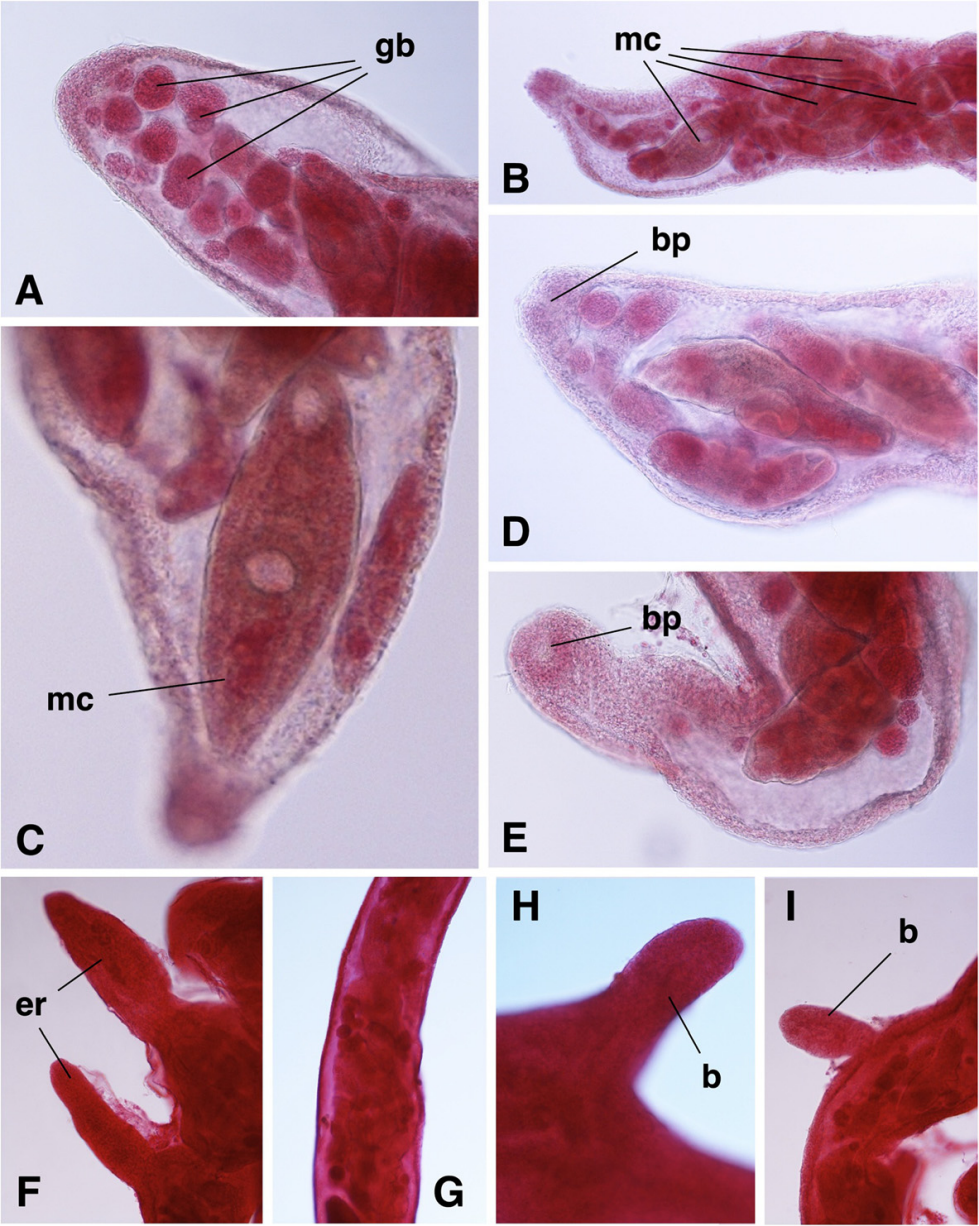
**Microscopic features of the sporocysts of *Renylaima capensis* .A**) numerous germinal balls (gb) inside tip of sporocyst ramification; **B**) numerous mature cercariae (mc) approaching tip of sporocyst branch; **C**) acaudate mature cercaria (mc), close to sporocyst branch birth pore, showing differentiated gonads and male genital duct; **D**, **E**) birth pore (bp) at the tip of terminal branch in lateral and apical views, respectively; **F**) two early ramifications (er) deriving from main sporocyst branch; **G**) sporocyst branch showing numerous germ balls and developing cercariae inside; **H**, **I**) small buds (b) developing from main sporocyst branch.

**Figure 4:**
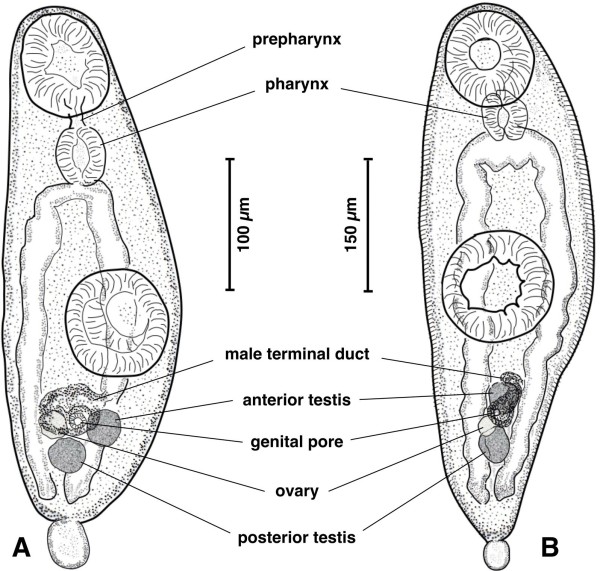
**Cercaria and metacercaria of *Renylaima capensis.* A**) caudate mature cercaria found inside a sporocyst infecting *Ariostralis nebulosa* in ventral view, showing prepharynx, differentiation of gonads, male terminal duct and ventral genital pore; **B**) caudate metacercaria from the kidney of *Ariopelta capensis* in ventral view, showing pronounced tegumental spinulation, gonads, male terminal duct and ventral genital pore. Scale bars: A = 100 μm; B = 150 μm.

**Figure 5:**
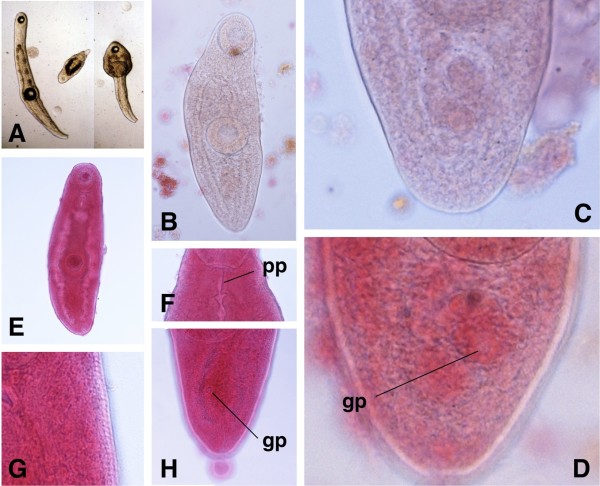
**Cercariae and metacercariae of *Renylaima capensis.* A**) photomicrographs showing living cercariae from sporocysts infecting *Ariostralis nebulosa*; note extension (left) and contraction (right) capacities in the same acaudate cercaria and small developing immature cercaria (centre) with caeca not yet reaching final part of body and terminal tail; **B**) acaudate mature cercaria from inside a sporocyst in ventral view; **C**) mature cercaria from a sporocyst showing absence of postacetabular rounded undifferentiated genital primordium typical of brachylaimids, but evident differentiation of genital structures; **D**) mature cercaria from a sporocyst showing ventral genital pore (gp); **E**) acaudate metacercaria from *Ariopelta capensis* in ventral view; **F**) long prepharynx (pp) in specimen of metacercaria in ventral view; **G**) tegumental spinulation in a metacercaria in ventral view; **H**) ventral genital pore (gp) in caudate metacercaria in ventral view.

Amongst these five pulmonates, sporocysts of the branched brachylaimed type (Figures 2A-D, 3A-I) containing cercariae (Figures 4A, 5A-D) were only found in *Ariostralis nebulosa*. Unencysted metacercaria (Figures 4B, 5E-H) resembling those previously described for various *Brachylaima* species were only found in *Ariopelta capensis*.

### Sporocysts

Non-cercariogenous mother sporocysts, similar to those described in other brachylaimid species [20-23,51] were never found in the slug specimens dissected. However, the existence of a first generation of non-cercariogenous sporocysts of a short duration cannot be excluded.

Branched, cercariogenous sporocysts of the brachylaimid type (Figure 2A-D) were frequently found in the slug *Ariostralis nebulosa*. These sporocysts were attached to the hepatopancreas, body wall, pallial floor and even to the genital system. The lumen of the branched tubular structures of the sporocysts contain germ balls of various degrees of development, developing cercariae and fully mature cercariae (Figure 3A-E, G). Those closest to the terminalia of the sporocyst branches were continuously retracting and extending their bodies. These extensions caused a stretching of the apices of the branches. Although no cercariae were observed to escape from the sporocysts, the presence of a birth pore, clearly visible at the apices of several terminal branches, suggest that they may be able to do so.

When comparing these sporocysts to cercariogenous sporocysts of other brachylaimids [14,16,17], two aspects should be highlighted. First, there is no ejection chamber at the tip of a terminal branch basal to the birth pore (Figure 3A-E); in *R. capensis*, the maximum width of the terminal part of the branches varies pronouncedly between 160.8 μm and 482.5 μm (mean 304.3 μm), with an inner continuous lumen without constrictions, and the tip of the branches shows a width of 80.4-126.3 μm (mean 105.6 μm) at the level of the birth pore. Secondly, lateral budding at the surface of these terminal branches appears only very rarely (Figure 3F-I), so that such terminal branches are relatively very long. No birth pore could be observed in these small lateral budding branches, which suggests that it may gradually develop with branch growth.

### Cercariae

Living cercariae extracted from the sporocysts vigorously extend and retract their bodies resulting in their length varying between less than 230 μm and up to 900 μm, with their shape also changing pronouncedly (Figure 5A). Once fixed, the body of mature cercariae is 330.0-436.6 μm long and 139.0-20.6.8 μm at maximum width. The measurements of mature cercariae are noted in Table1.

**Table 1:** **Morphometric characteristics of cercariae and metacercariae of *Renylaima capensis* **

**Larval stage**	**Cercariae**			**Metacercariae**		
**Intermediate slug host species**	**Ariostralis nebulosa**			**Ariopelta capensis**		
**No. larvae studied**	**n = 10**			**n = 10**		
	**E.V.**	**X**	**SD**	**E.V.**	**X**	**SD**
Length	330.0-436.6	392.9	40.4	528.5-694.4	604.6	52.1
Maximum width	114.9-166.3	142.0	18.6	139.0-206.8	176.7	20.0
Oral sucker (OS)	68.9-85.9/62.9-80.4	76.5/67.5	6.4/5.7	82.6-108.4/76.9-103.4	92.4/87.7	9.3/8.0
Ventral sucker (VS)	80.4-94.9/68.9-85.9	86.5/78.1	6.3/5.2	96.9-131.3/91.2-119.9	115.7/106.5	13.0/12.6
Sucker ratio (OS/VS)	0.62-0.85	0.73	0.07	0.57-0.83	0.68	0.08
Prepharynx (length)	0.0-12.2	5.4	5.5	0.0-37.0	16.3	11.4
Pharynx (length/width)	34.4-39.9/26.4-34.4	36.6/32.3	2.7/3.2	39.9-57.4/31.3-45.9	45.0/38.6	5.4/4.3
Anterior testis (T1)	22.0-28.6/34.0-34.8	33.5/26.8	2.1/5.6	25.6-57.0/18.0-37.0	40.3/29.6	10.7/6.5
Posterior testis (T2)	22.9-34.4/20.0-28.6	28.9/23.9	4.6/3.0	34.2-48.4/22.8-37.0	39.5/30.0	5.0/4.9
Ovary	22.0-28.6/17.0-22.9	25.2/20.4	2.9/2.7	25.6-31.3/19.9-25.6	28.9/23.2	2.1/2.1
Distance OS-VS	85.9-137.8	112.3	21.2	126.3-245.1	177.1	35.0
Distance VS-T1	17.5-40.0	28.6	8.0	37.0-71.2	53.8	13.3
Tail (length/width)	22.9-35.4/25.9-28.4	30.9/26.9	6.9/1.3	28.5-37.0/27.1-37.0	33.1/30.8	2.7/3.4

Meaurements in μm. *E.V*. = extreme values; *X* = mean values; *SD* = standard deviation.

These cercariae are of the cercariaeum type (brevicaudate cercariae), similar to the so-called microcercous type in aquatic cercariae, but lacking a stylet (Figure 4A). Its morphology follows the general pattern of brachylaimid cercariae, with well developed caeca up to almost the posterior end of the body and excretory canals of the stenostoma type.

There are, however, several species-specific features, such as the very fine spinulation regularly distributed all over the body almost up to the posterior end of the body, the postequatorial location of the acetabulum, a sucker ratio of less than 1 compared to 1 in the adult stage [39], a prepharynx sometimes present (Figure 4A), and a very small tail not always present (Figures 4A5B), even in intrasporocystic mature cercariae (Figure 3C). But the most exceptional characteristic of the mature cercariae of *R. capensis* concerns the genital structures, located in the postacetabular region between the caecal ends and at a short distance from the acetabulum of 22.9-34.4 μm (mean 26.7 μm). This genital complex appears already divided in mature cercariae inside the sporocyst (Figure 3C), with a well visible anteriorly situated terminal part of the male duct ending in a small ventral genital pore, and a genital anlagen already fragmentated into the three gonads (Figures 4A5C, D).

### Metacercariae

Unencysted metacercaria are found in the kidney of the slug *Ariopelta capensis*. In a heavily infected specimen, an almost solid white mass was found when opening the slug’s kidney. This whole seemingly solid mass filled the kidney and proved to consist of a large number of metacercariae.

The body of the metacercaria is covered with spines in the same pattern as that in the adult specimens occurring in the shrews [39]. Tegumental spines are visible up to the posterior extremity of the body and their length is 7.1-8.5 μm at the level of pharynx and preacetabular region. Metacercariae structurally also resemble the above described cercariae. Fixed metacercariae are 528.5-694.4 μm long and 139.0-206.8 μm at maximum width (Figure 4B), that is, only slightly larger than cercariae. Measurements of fixed metacercariae are given in Table1.

The sucker ratio and the appearance of a prepharynx duct are maintained as in cercariae, but the acetabulum tends to be more equatorial in location due to the enlargement of the postacetabular region. A tail, wider at mid-length than at its base, appears to be almost always present (Figure 4B), which suggests that its absence in a few specimens (Figure 5E) may be due to its loss during the extraction, staining and mounting processes.

As typical in brachylaimid metacercariae, the three gonads appear already well differentiated (Figure 4B), similarly as seen in the well individualised final genital ducts of both male and female systems. The genital pore is clearly visible in a non-protruding atrium, located ventrally (Figures 4B5H), in a location similar to the one in the adult stage [39]. This larval form indicates that the normal brachylaimid life cycle with two intermediate pulmonate hosts also applies here.

### Definitive mammal host

Adults of *R. capensis* were very often found in shrews of the species *Myosorex varius* (Figure 1A). It seems evident that the brachylaimid adults hosted by the shrews develop from the aforementioned larval stages harboured by these slugs. This is moreover supported by the fact that adult trematodes were never found in any of the other vertebrates living at this locality. These other vertebrates are two rodents, the vegetarian *Otomys irroratus* and the Cape spiny mouse *Acomys subspinosus*, and two amphibians, the toad *Breviceps acutirostris* and the frog *Arthroleptella landdrosia*. No slug-eating birds were ever observed in the locality. The very dense and impenetrable vegetation would make such a predation almost impossible in any case.

### Final microhabitat and intraorganic migration

The final microhabitats of *R. capensis* are the kidneys and ureters of the shrews, kidney pelvis and calyces in light infections and also kidney medulla and cortex in heavy infections. In heavy infections both kidneys and ureters can be infected and in one such a case up to 43 individuals were recorded from only one of the ureters while its corresponding kidney contained more than 30 individuals.

A morphological study of specimens found in shrew kidneys showed that 11.1% of the flukes were monotesticular forms only having a single postovarian testis.

The microhabitat of this brachylaimid inside the urinary system implies a transmission route through egg shedding with urine. Interestingly, fluke eggs fitting the characteristics (yellow-brown, symmetrical, oval, operculate and containing well developed embryos) and measurements (22.6-34.0 μm long and 14.7-20.6 μm wide) of *R. capensis* eggs were found on shrew’s droppings.

Unfortunately, migrating flukes were not found in shrew dissections. However, a young individual found attached to the outer surface of the shrews kidney, and other young individuals in some cases seen just under the surface of the kidney, are worth mentioning. Additionally, very young and small individuals, resembling the metacercaria found in the slug *Ariopelta capensis*, were also found amongst fully developed individuals in the kidneys and ureters. They present different stages of development. The smallest individual of these only measured 930 μm in length. The three primordial gonads and vitelline glands can be clearly distinguished and a developing uterus can be observed as a small looped but empty duct in the area posterior to the acetabulum in these small individuals.

### Epizootiology

#### Habitat, rainfall and humidity

Rainfall data covering a period of several years (1978–2011) were obtained from the meteorological station of the rain gauge at Disavlei (34° 00′ 32.80¨ S, 19° 00′ 51.95¨ E), the closest one to the locality where *R. capensis* is found (Table2). These data show that the precipitation in this area is very high throughout the year. The precipitation for the years 1994 to 2007 shows a relatively dry period in spite of two or three years of normal rainfall.

**Table 2:** **Precipitation (in mm) measured in the meteorological station of the rain gauge at Disavlei (34° 00′ 32.80¨ S, 19° 00′ 51.95¨ E), the closest one to the locality where *Renylaima capensis* is found, during the year period of 1978–2011**

**Year**	**January**	**February**	**March**	**April**	**May**	**June**	**July**	**August**	**September**	**October**	**November**	**December**	**TOTAL**
1978	210,8	75,3	57,4	308,9	169,2	109,8	201,7	865,6	430,2	300,4	165,0	289,6	3184
1979	90,8	87,6	126,5	123,0	275,4	695,0	254,7	288,5	209,6	410,2	123,0	19,7	2704
1980	163,5	130,9	41,8	436,9	375,7	419,5	83,2	368,9	162,7	186,5	288,6	236,0	2894
1981	333,0	33,5	153,3	204,0	65,5	322,8	504,2	704,5	457,5	93,5	247,4	130,0	3249
1982	183,0	40,1	103,5	323,0	208,0	280,7	348,0	230,9	221,7	273,6	171,9	226,2	2611
1983	57,8	191,8	129,8	57,5	828,4	860,0	362,1	176,0	381,0	85,5	77,3	70,4	3278
1984	63,0	66,0	173,8	167,0	723,2	89,5	716,8	221,0	420,0	301,7	79,2	307,5	3329
1985	182,5	167,3	340,9	219,5	236,4	580,0	403,9	382,8	316,5	144,5	172,0	100,0	3246
1986	123,5	118,4	130,5	423,0	278,9	568,0	460,0	733,9	247,0	81,0	109,9	66,5	3341
1987	191,5	90,0	84,7	198,6	436,5	458,9	485,5	374,9	355,0	115,5	102,0	227,5	3121
1988	17,3	25,0	70,0	351,2	234,6	361,9	437,2	504,5	?	225,9	80,9	109,0	2418
1989	5,5	88,8	259,0	348,3	312,7	369,0	420,7	401,7	688,2	273,5	184,9	51,0	3403
1990	75,0	167,9	64,5	737,8	158,6	312,4	868,3	452,3	183,6	62,0	138,0	89,2	3310
1991	85,0	57,9	62,8	131,0	490,4	605,5	919,4	69,8	411,0	219,1	54,7	43,5	3150
1992	33,8	122,9	148,3	376,5	482,0	847,0	451,3	362,5	358,7	431,3	147,5	56,9	3819
1993	39,7	103,0	19,5	908,2	573,0	602,4	881,0	293,0	82,5	51,5	89,0	118,0	3761
1994	108,4	55,9	33,0	200,6	195,2	870,0	281,6	209,5	224,0	121,4	90,0	86,9	2477
1995	110,4	25,0	87,0	158,2	306,0	390,0	680,0	408,4	138,5	350,0	45,4	263,0	2962
1996	20,5	136,7	76,0	166,4	166,0	470,0	410,0	0,0	841,1	539,0	430,0	213,0	3469
1997	135,0	21,5	45,0	164,0	225,0	687,0	151,8	415,0	300,0	207,0	427,0	0,0	2778
1998	206,0	43,0	128,0	246,0	625,0	0,0	790,0	270,0	180,0	0,0	370,0	315,0	3173
1999	9,0	18,0	17,0	187,0	280,0	300,0	436,0	200,0	0,0	180,0	123,0	31,0	1781
2000	105,0	94,0	96,0	57,0	0,0	695,0	240,0	0,0	800,0	107,0	57,0	95,0	2346
2001	24,0	18,5	7,0	0,0	820,0	100,0	0,0	0,0	0,0	1040,0	0,0	93,0	2103
2002	360,0	57,0	0,0	0,0	720,0	558,0	0,0	720,0	0,0	506,0	100,0	75,0	3096
2003	71,0	65,0	280,0	160,0	0,0	280,0	0,0	840,0	0,0	460,0	8,0	0,0	2164
2004	15,0	330,0	0,0	540,0	45,0	0,0	0,0	0,0	840,0	228,0	0,0	0,0	1998
2005	210,0	164,5	0,0	0,0	0,0	991,0	328,0	520,0	0,0	0,0	0,0	0,0	2214
2006	58,0	0,0	58,0	23,5	51,0	334,0	297,0	400,0	0,0	228,0	140,0	100,0	1690
2007	8,0	12,9	174,5	169,0	280,0	612,0	640,1	580,0	68,0	0,0	640,0	0,0	3184
2008	230,0	0,0	182,0	0,0	513,0	467,0	638,0	310,0	612,0	52,0	365,0	60,0	3429
2009	?	?*	39,0	160,0	863,0	623,5	248,0	620,0	304,0	?	?	?	3554
2010	14,5	68,0	280,0	110,0	655,5	304,0	230,0	271,0	175,0	205,2	156,0	106,4	2576
2011	21,0	23,5	3,0	212,8	180,0	761,0	87,0	324,0	186,0	140,0	190,0	106,4	2235

? = measurement could not be performed in this month because the gauge was broken.

* = a fire occurred in early February 2009 which pronouncedly damaged the vegetation in the habitat of *R. capensis*.

TOTAL = total rainfall for each year; a graphic comparison of yearly rainfall data available since 1945 from that meteorological station shows that the period 1999–2006 was the most dry period.

In this area, winter rains from May up to September-October are normal. Thus, most of the precipitation measured in the summer period (from October to April) is from mist, which is brought in to the mountain tops by the South-Eastern wind. The locality of *R. capensis* being at the top end of a ravine that faces in a South Western direction also regularly receives mist in summer via the South Western wind. Therefore, the transmission area of this brachylaimid presumedly has a slightly higher precipitation than the closely located area at Disavlei, as it receives mist from two wind directions.

To understand the rainfall data, it should also be considered that although 2008 and 2009 were relatively good rainfall years, after a long dry period, a fire that occurred in early February 2009 damaged the vegetation in the habitat of *R. capensis*. This fire had a significant influence as it burnt away much of the very thick Restionaceaea vegetation and especially the rotting undergrowth at the bases of these plants. The latter when intact, acts as an efficient sponge to retain the water and keep the area very moist. It is also the nesting habitat for the slug *Ariopelta capensis*. After the fire it was mostly burnt away and much of the water simply flowed away with the effect that the habitat was drier than normal. The previous dry period obviously did put stress on the vegetation so that more dry dead plant material accumulated. Consequently, when the fire occurred it could burn with more intensity.

#### Prevalences and intensities in the slugs

*Ariostralis nebulosa* is readily found throughout the whole year. During the years 1982–2011, a total of 38 specimens of *A. nebulosa* were infected with sporocysts among the 263 specimens collected (Table3). The aforementioned perturbation of the habitat of *R. capensis* by the fire, which occurred in February 2009, should be taken into account. Before that fire, from 198 *A. nebulosa*, 34 were infected by sporocysts (mean 17.2%), with high prevalences in given years, whereas after the fire infection disappeared up to 2011 when this infection rate seemed to recover. A seasonal analysis of data before the fire indicate that sporocyst infection is mainly detected from late autumn (April) to early spring (September) and only sporadically in summer time (late October to March). This suggests a seasonality in the transmission dynamics of the parasite. Whether sporocysts are so pathogenic that infected slugs are not able to survive until the summer or are perhaps more easily predatable remains a question for future research.

**Table 3:** **Prevalences of infection of the first intermediate slug species *Ariostralis nebulosa* by cercariogenous sporocysts of *Renylaima capensis* according to years and months**

**Year of collection**	**Month (and day) of collection**	**No. of slugs collected**	**No. infected with sporocysts**
1982	April 23	10	4 (40%)
	July 19	4	1 (25%)
1983	March 2	2	0 (0%)
	April 23	4	1 (25%)
2004	October 19	4	0 (0%)
1983-2006 period	March-September	127	26 (20.5%)
	October-February	47	2 (4%)
2009	in February, a fire burnt a large part of the habitat		
2009	May 9	3	0 (0%)
2010	February 10	0	--
	April 26	21	0 (0%)
	October 11	5	0 (0%)
2011	February 22	15	1 (6.6%)
	May 11	21	3 (14%)
	July 14	7	0 (0%)
**Total**		**263**	**38 (14.4%)**

Contrary to *A. nebulosa*, *A. capensis* is mostly found during the hotter months, the October-March period (nests of eggs of this slug species were found in January, that is, in midsummer). During the years 1978–2011, only 3 specimens of *A. capensis* were infected with metacercariae among the 77 specimens collected (Table4). Before the fire, only two (collected in April and May) from 49 specimens carried metacercariae (mean 4.0%), surprisingly lower than the prevalence of sporocysts in *A. nebulosa*. The third specimen infected by metacercariae, collected in 2011 (after fire) was also found in May. This suggests a concentration of second intermediate slug infection by metacercariae in the colder months. Additionally, the pronouncedly lower number of *A. capensis* collected indicates lower population densities of the second intermediate slug when compared to the first intermediate one. With regard to infection intensity, while only one metacercaria was found in one *A. capensis*, the other two specimens showed relatively massive infections.

**Table 4:** **Prevalences and intensities of infection of the second intermediate slug species *Ariopelta capensis* by metacercariae of *Renylaima capensis* according to years and months**

**Year of collection**	**Month (and day) of collection**	**No. of slugs collected**	**No. infected with metacercariae**	**No. of metacercariae per slug**
1978	May 18	4	1 (25%)	1
1982	January 8	3	0 (0%)	-
	February	1	0 (0%)	-
	April 23	4	0 (0%)	-
	July 19	0	--	-
1983	March 2	5	0 (0%)	-
	April 23	4	1 (25%)	>70
1984-1985	month?	12	0 (0%)	-
	June 16	0	--	-
1986	May 10	5	0 (0%)	-
1994	July 17	0	--	-
1997	October 10	2	0 (0%)	-
	November 1-7	7	0 (0%)	-
2004	October 19	0	--	-
	December 16	0	--	-
2005	April 28	0	--	-
2006	March 14	0	--	-
	May 13	2	0 (0%)	-
	November 30	0	--	-
2009	in February, a fire burnt a large part of the habitat			
2009	May 9	3	0 (0%)	-
2010	February 10	0	--	-
	April 26	3	0 (0%)	-
	October 11	6	0 (0%)	-
2011	February 22	0	--	-
	May 11	12	1 (8.3%)	> > 100
	July14	4 (young)	0 (0%)	
**Total**		**77**	**3 (3.9%)**	

#### Prevalences and intensities in the shrews

The shrew species *Myosorex varius* appears to be active mainly from October to May. During the years 1983–2010, a total of 21 specimens of this shrew species were infected with adults of *R. capensis* among a total of 29 specimens collected, giving a prevalence of 72.4% during this long period (Table5). In many years, all specimens captured proved to be infected, and when not all were infected, yearly prevalences found were usually very high. Additionally, intensities also proved to usually be very high, sometimes even higher than 50 adult flukes per shrew, with an apparent decline after the fire occurred in February 2009.

**Table 5:** **Prevalences and intensities of infection of the definitive host species *Myosorex varius* by adults of *Renylaima capensis* according to years and months**

**Year of collection**	**Month (and day) of collection**	**No. of shrews collected (20 traps set)**	**No. of shrews infected**	**Intensity (no. of flukes/shrew)**
1983	April 4	1	1 (100%)	>50
	June 16	0	--	-
1986	May 10	3	3 (100%)	>50 +37 + >50
1994	July 17	0	--	-
1997	October 10	1	1 (100%)	>50
	November (1–7)	2	2 (100%)	>50 + >50
2004	October 19	0	--	-
	December 16	3	1 (33%)	45 + 0 + 0
2005	April 28	4	3 (75%)	33 + 16 + 13 + 0
2006	March 14	3	3 (100%)	9 + 1 + 5
	May 13	4	3 (75%)	11 +7 + 24 + 0
	November 30	2	1 (50%)	57 + 0
2009	in February, a fire burnt a large part of the habitat			
2009	May 9	0	--	-
2010	February 10	2	2 (100%)	16 + 6
	April 26	4	1 (25%)	2 + 0 + 0 + 0
**Total**		**29**	**21 (72.4%)**	**>527**

### Mitochondrial DNA *cox*1 sequences

DNA sequences obtained for the mtDNA *cox*1 gene fragment were of a length of 437 nucleotides. All sporocysts, cercariae and adults showed the same sequence, with a composition of 64.75% AT. However, the metacercariae available for sequencing, coming from only one *Ariopelta capensis* slug individual, showed three mutations (6.86% divergence) when compared to the sequence of the other developmental stages: (i) C in metacercariae and G in the other stages in position 80, (ii) T/G respectively in position 118, and (iii) C/T respectively in position 373. The AT content of the metacercarial sequence was also 64.75%.

The provisional haplotypes R.cap-cox1a and R.cap-cox1b have been ascribed to the sequences found in sporocysts, cercariae and adults on one side, and metacercariae on the other side, respectively. Their respective accession number codes are [EMBL: HE663453] and [EMBL: HE663454].

**Figure 6:**
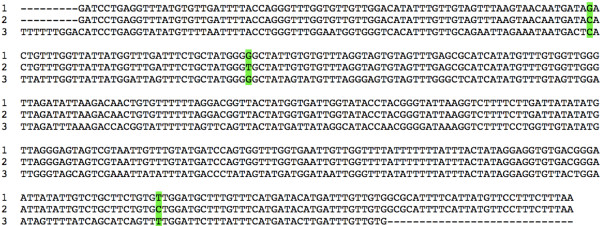
**Nucleotide alignment of the mitochondrial DNA *cox* 1 gene sequences.** Line 1: R.cap-cox1a haplotype sequence obtained from sporocysts, cercariae and adults of *Renylaima capensis*; Line 2: R.cap-cox1b haplotype sequence obtained from metacercariae of *Renylaima capensis*; Line 3: last part of the 897-bp-long fragment from *Glaphyrostomum* sp. [GenBank: FJ713138] [52]. Nucleotides distributed according to 90 positions per line. Three mutation differences between the two haplotypes of *R. capensis* marked in green.

When using the BLASTN programme, homologies were found with several *cox*1 fragments of other digenean species of which the same or very similar fragment is available in databases. The species that provides the highest query coverage (94-96%) in a total score of 231–259 bp compared is *Schistosoma japonicum*. A specific search using the nucleotide database of the GenBank showed that very few sequences of brachylaimids are available. Among them, the only *cox*1 sequence available is the 897-bp-long fragment of this gene from *Glaphyrostomum* sp. [GenBank: FJ713138] [52]. In a comparison between *Renylaima* and *Glaphyrostomum* haplotypes, by means of a 420–11 (= 409) bp-long alignment, the result was congruent despite the numerous mutations that appear, with a total of 78 variable positions (19.07% divergence) (Figure 6).

The resulting protein fragments from *R. capensis* are both composed of 145 aminoacids and are available in the EMBL database under the same accession numbers. An alignment comparison between them shows that of the three nucleotide mutations two give rise to amino acid changes whilst the third one is silent.

## Discussion

### Life cycle

A total of 34 years work in the small area where *R. capensis* occurs, leads to several conclusions: (i) this fluke species appears to be specific for *M. varius*, as no brachylaimid has ever been found in any of the other vertebrates inhabiting the same area; (ii) the slug *Ariostralis nebulosa* is the first intermediate host of this fluke species, as branched, cercariogenous sporocysts resembling those typical of brachylaimids [14,16,24,25] were frequently found but only in this slug among the five terrestrial mollusc species inhabiting the area; this means that this fluke is highly specific at first intermediate host level; (iii) *Ariopelta capensis*, the only other slug species present in the same area, appears to be the only second intermediate host species, as brachylaimid metacercariae resembling immature juveniles found in the shrews were only found in this mollusc. Although no intracellular early mother sporocyst stage within hepatopancreatic cells [23] was observed nor any sporocystogenous first generation sporocysts [19-22], the existence of such stages cannot be *a priori* denied.

The sequencing results verify that the sporocysts, cercariae, metacercariae and adult stages belong to the same species and confirm this life cycle. The mere three mutations found in the metacercariae infecting an *Ariopeltis capensis* slug individual may be considered as intraspecific genetic variability within the local area, according to the well known evolutionary rates of the mtDNA *cox*1 gene in invertebrates and its usefulness for inter- and intrapopulation diversity studies in general [41]. This agrees with the three-host life cycle pattern of Brachylaimidae. The observation that the shrew *M. varius* preys on both species of slugs supports this view.

However, results of the several years of field work indicate that prevalence and intensity data do not appropriately fit the above statement:

 a) in brachylaimid species in general, the prevalences of the first intermediate snail host by cercariogenous sporocysts are always pronouncedly low [14,16,17], whereas in the case of *R. capensis* the prevalence is pronouncedly higher, mainly in autumn-spring;

 b)in brachylaimids, prevalences and intensities of the second intermediate snail host by metacercariae are usually very high, even up to 100%, and high infection burdens of up to more than 100 metacercariae occur in the main second snail host [14,16,17]; on the contrary, in *R. capensis*, the prevalence is strikingly low; this may be explained by the second intermediate slug host not being readily available in winter and thus allowing second intermediate slug infections only very rarely;

 c) the high prevalences and intensities of *R. capensis* in the shrews do not correspond with the very low prevalences (and sometimes apparently also intensities) of metacercariae in slugs, nor with the second intermediate slug population densities in the area;

 d) the adult stage does not show an egg production capacity higher than that of other *Brachylaima* species (similar body size, similar size of the gonads, similar uterus extent); the fluke prevalence in the shrews (72.4%) is, however, higher than those usually found for intestinal *Brachylaima* species in their definitive host species: for instance, 56.7-64.3% in one host species and 21.4-60.0% in another host species for *B. ruminae*[18], or 38.5-66.7% for *B. mascomai*[53]; moreover, *M. varius* shrew densities are not as high as to argue that a higher egg dissemination ability could be the cause for higher infection rates of the first intermediate slug species.

From the point of view of the r/K selection concept, ecological studies on the compared fitness of the life cycle strategies of Brachylaimidae in general show that these trematodes follow a compensation pattern within the different transmission phases [18]. According to such a rule, the surprising prevalence and intensity data at the levels of first and second intermediate hosts, markedly opposite to what is found in other brachylaimids, can be understood, although the route of transmission may still remain an open question.

The question immediately arises about the possibility of direct definitive host infection by ingestion of mature cercariae still inside sporocysts carried by the first intermediate mollusc host. Although definitive host infection by mature cercariae resulting in the development of mature adults, without being exposed to metacercariae, could not be proven, observations in three brachylaimid species suggest that this could be possible. In the species *Z. spearei*, juvenile specimens presenting a cercarial tail were found in the urinary bladder of the toad *Bufo marinus* and it was concluded that this may be indicative of the possibility that the cercaria in the first intermediate host could be directly infective to the final host. This theory is further supported by the statement that in the smallest of these individuals the genital system was only represented by a single primordium [29]. In *Brachylaima ruminae*, such a cercarial tail was also found in several juvenile specimens from the intestine of garden doormice *Eliomys quercinus* (Valero and Mas-Coma, unpublished data). In *D. frontalis*, a cercarial tail was found in a juvenile fluke [37] as well as in three gravid fluke specimens (Valero and Mas-Coma, unpublished data) from the nasal sinuses of the same garden dormouse species. However, a cercarial tail such as this appears to be sometimes retained for a period after having penetrated the second intermediate snail host [54], so that it cannot be ruled out that tailed adults may also derive from tailed metacercariae infecting a second intermediate snail host. The numerous tailed metacercariae found in one *Ariopelta capensis* indicate that this phenomenon may also take place in *R. capensis*.

Moreover, the presence in mature cercariae of *R. capensis* of a genital complex already divided, with visible terminal male duct ending in a small ventral genital pore, and a genital anlagen already forming the three gonads, should be highlighted. Such a precociousness is not typical of Brachylaimidae. In species of this family, only a rounded, postacetabular, undifferentiated genital primordium appears in the mature cercariae [14,16,17]. This suggests that mature cercariae may develop sufficiently inside the sporocyst as to become infective for the definitive host (i.e., do not need further genital maturation in a second intermediate host). Additionally, the very small increase in fluke size during their transmission through the three hosts (length of mature cercariae inside the sporocyst, metacercariae in slug kidney and smallest adult in the shrew of 330–436 μm, 528–694 μm, and 930 μm, respectively), suggests that a develpment transit in a second intermediate host may not be necessary. In other terms, a larger size development of metacercariae in the second intermediate slug host could become an impediment for a subsequent successful intraorganic migration within the shrew.

The capacity of self-infection of the first intermediate mollusc host individual with metacercariae shed by the sporocysts harboured by the same molluscan individual also implies the reduction of the life cycle from a three-host pattern to a two-host pattern. Such a capacity is apparently related to an adaptation strategy of the characteristics of both intermediate mollusc host and habitat environment [18]. Such self-infection seems to be prevented by a kind of premunition in species such as *B. ruminae*, which is consequently an obligatory three-host brachylaimid [16]. In the species *D. frontalis* inhabiting dry habitats, 90.6-100% of the snails bearing sporocysts also harbour metacercariae in the pericardium while only 19.9-40.0% of the snails, of the same species, lacking sporocysts carry metacercariae [14]. Similarly, in a *P. pericardicum* inhabiting wet habitats, the relative figures are 50.0% and 39.2% [17].

In another brachylaimid, *Serpentinotrema laruei* (= *Postharmostomum laruei*; = *P. helicis*) [19-21,55-57], the discovery of a precociously developed metacercaria within a sporocyst [58] also indicates the secondary possibility for a two-host life cycle. In Brachylaimidae, however, the genus *Parabrachylaima* presents a two-host life cycle, as the adult stage fully develops to maturity and egg laying within a terrestrial snail [32]. Progenesis within a former second intermediate snail host has been the interpretation used to justify its inclusion into the subfamily Brachylaiminae [33]. All in all, Brachylaimidae appear to be a versatile group where the elimination of one or another of the typical hosts in the life cycle might be possible. It thus seems as although a triheteroxenous life cycle is indicated for the new South African fluke species, the possibility of the second intermediate host being eliminated in current transmission cannot be ruled out.

A reduction from a three-host life cycle to a two-host life cycle has been described in species in other families included within Brachylaimoidea, namely Hasstilesiidae from mammals, and Leucochloridiidae and Leucochloridiomorphidae from birds. In all of them, metacercariae develop in branched sporocysts infecting snails (terrestrial snails in hasstilesiids and leucochloridiids, and aquatic snails in leucochloridiomorphids) [13,15,59-61]. Leucochloridiidae and Leucochloridiomorphidae seem to be morphologically and biologically distant from the South African fluke, but hasstilesiid species of the genus *Strzeleckia* from the intestine of marsupials appear to be not as distant morphologically [62]. Another genus, *Michajlovia*, comprises parasites which infect the intestine of passerine birds and whose life cycle is still unknown. *Michajlovia* adults present a ventral genital atrium just posterior to the gonads or in the region of the posterior testis. *Michajlovia* is included in Brachylaimoidea as *incertae sedis* although close to Leucochloridiidae and Leucochloridiomorphidae [31,63], and also shows several similarities with the South African fluke. Except for the extent of vitellaria, the morphological similarities of *Michajlovia* with the panopistine genus *Dasyurotrema*, whose type species *D. mascomai* infects the alimentary tract and associated organs of marsupials [64], are evident.

### Fluke transmission, monotesticular forms and their origin

The not uncommon monotesticular forms of *R. capensis* adults merit an additional analysis to elucidate fluke transmision. In these curious adults, the single postovarian testis is relatively much larger and elongated than either the anterior or posterior testes of the bitesticular specimens [39].

The phenomenon of neoteny, as previously defined [65], seemingly applies to these individuals seeing that the testis matures in the adult without the larval genital anlagen having separated into two independent testes beforehand. This fits the concept of cercariae having the capacity to infect the definitive host, as the genital primordium in brachylaimid cercariae does not differentiate into the three separate gonads prior to the development of the mature metacercaria within the second intermediate snail host [14,16,17]. Monotesticular forms of *R. capensis* thus also support a direct transmission from first intermediate snail host to definitive host, as such forms may derive from intrasporocystic cercariae already infective but with division of the genital primordium still not complete.

Monotesticular specimens have also been found, but rarely, in other brachylaimid species. Interestingly, modern brachylaimid species such as *Brachylaima ruminae*, it is always the posterior testis that is absent in the monotesticular forms. Contrarily, in presumably archaic brachylaimids such as *Ityogonimus ocreatus* from moles (Talpidae insectivores) and *Dollfusinus frontalis* from hedgehogs (Erinaceidae insectivores) and dormice (Glirimorph rodents), it is always the anterior testis which is absent. Noteworthy is that certain adult specimens of *D. frontalis* show a rare triangular arragement of the gonads in which the ovary appears anterior to both testes (Valero and Mas-Coma, unpublished data), thus remimiscent of the gonad arrangement typical of *Zeylanurotrema*.

A strong argument to support a direct cercarial origin for monotesticular specimens is found in the monorchid species *Parabrachylaima euglandensis*. In this peculiar brachylaimine, the progenetic adult stage fully develops to maturity and egg laying in a terrestrial snail, and shows only one postovarian, sacculate testis with a pair of anterior projections from which the two respective vasa efferentia arise [32]. Additional to its peculiar life cycle, the following features of *Parabrachylaima*, however, rules out a close relationship with the South African fluke: (i) both suckers close together in the anterior part of the body; (ii) caeca unequal, with right caecum terminating in middle third of body; (iii) very long excretory vesicle reaching anteriorly to level of acetabulum; and (iv) cirrus pouch present and including a poorly developed cirrus.

If a definitive-host-infection capacity is accepted for intrasporocystic mature cercariae of *R. capensis*, massive infections of the shrews by adult flukes are easily explained (one or a very few sporocyst-carrying slugs ingested would be sufficient), but on the contrary a question mark is posed by the shrews infected by only a few adult flukes. Such low infections could be the consequence of (i) shrews swallowing sporocysts including only a few mature cercariae at that moment (i.e., all other intrasporocystic cercariae present were still immature at that moment, not able to migrate inside the shrew’s body and hence eliminated digestively after ingestion), (ii) most cercariae may get lost in the definitive host infection, most probably expelled by the shrew with their faeces; thus, the massive sporocyst infection of the first intermediate slug host and the very long sporocyst branches filled by mature cercariae could be interpreted as a fluke strategy to mitigate the great losses; (iii) a crowding effect not allowing the penetration into the kidney and ureters by all migrating cercariae because of lack of microhabitat space, (iv) massive infections becoming too pathogenic for shrews which would thus be quickly eliminated from natural populations, and of course also (v) ingestion of slugs only carrying a few metacercariae. In addition to all this, an influence of innate and cellular immunity on establishment of infection may also play a role in such individual cases.

### Mitochondrial DNA *cox*1 gene sequence variability and transmission modalities

It is evident that the number of slugs and shrews, from which respective larval stages and fluke adults have been molecularly analysed, is insufficient to conclude significant results. Unfortunately conservation restriction laws will never allow the collection of a sufficient host number, mainly of *Ariopelta capensis*, to obtain sufficient amount of metacercariae from different slug individuals, to perform the necessary study, given the pronouncedly low metacercarial prevalences in this slug species.

The characteristics of the three mutations found in the metacercariae are surprising. In mtDNA coding genes, when intraspecific mutations appear they are mostly silent and only a small percentage give rise to amino acid changes [41]. In *R. capensis*, among three mutations only one is silent. Whether such a rare genetic characteristic may be interpreted as two different biological strains of *R. capensis* coexisting in the study area may always remain a question mark. But there is the temptation to suggest that there coexists a majority of haplotype R.cap-*cox*1a strain following a two-host life cycle modality with a less frequent R.cap-*cox*1b strain following a three-host life cycle modality. Such a parallel circulation of the two biological strains could be perhaps related to climatic seasonality, due to *Ariopelta capensis* not being readily available in winter.

### Intraorganic migration, adult microhabitat and egg shedding

The occurrence of *R. capensis* in the urinary system of a mammal host is a unique phenomenon for the family Brachylaimidae. Within Brachylaimoidea, only the species of *Zeylanurotrema* parasitising amphibians and reptiles show a similar microhabitat: *Z. lyriocephali* in the urinary bladder of the agamid lizard *Lyriocephalus scutatus* in Sri Lanka [38] and *Z. spearei* in the urinary bladder of the cane toad *Bufo marinus*[29]. However, there are fundamental differences between the urinary system of mammals and that of amphibians and reptiles. In mammals, the urinary system is isolated from the alimentary tract, so that a renal helminth following an oral way of infection unavoidably has to traverse tissues, then find the kidney and actively penetrate it. On the contrary, in lizards the urinary system opens into the cloaca forming a direct connection along which the parasite can migrate between the two systems. Similarly, in amphibians the urinary bladder is practically merely an evagination of the alimentary tract. Furthermore, the distinct anatomy of *Zeylanurotrema* having a very anterior, postbifurcal acetabulum, pretesticular, lobed ovary, opposite testes and terminal genital pore rules out any close relationships with *R. capensis*.

Within Brachylaimoidea, another additional exception presenting a similar microhabitat, although inside a definitive snail host, is the “progenetic” adult stage of *Parabrachylaima euglandensis*, which also develops in the lumen of the kidney sac [32].

That an active, tissue-traversing, intraorganic migration within the shrew takes place during its infection by *R. capensis*, is the only way to understand the observation of both (i) a young individual attached to the outer surface of the shrews kidney, and (ii) other young individuals in some cases seen just under the surface of the kidney which may be interpreted as immature flukes just penetrated and on their way to the deeper areas of the kidney and the ureters.

Concerning the intraorganic route followed by the infective stage to the kidney, the knowledge about such a life cycle phase in other renal helminths suggest that a probable intermediate migratory step through the liver could be envisaged. This is the case in the nematode *Dioctophyme renale*, a renal parasite of carnivores [66], as well as, interestingly, that of another trematode from European insectivores (shrews and moles) but also archaic glirimorph rodents (dormice), *Nephrotrema truncatum*[67,68]. In both cases, the infective stage crosses the intestinal wall and migrates through the general body cavity to penetrate the right hepatic lobes which cover the right kidney before penetrating the latter. Such a liver phase appears to be important from the trophic point of view [66]. Contrary to *N. truncatum* where the adult stage only infects the right kidney, in *R. capensis* the adult stages infect both kidneys and ureters without any apparent lateral preference [39]. This poses the question whether *R. capensis* migrates throught the choledoc duct up to the biliary ducts to finally reach both kidneys after crossing the distal parenchyma of the liver lobes. Such hypothesis is supported by the hepatic duct microhabitat of species of another close brachylaimid genus, namely *Scaphiostomum*[36], although a more simple migration only including intestinal wall crossing and direct migration through the general body cavity to enter the two kidneys of the shrew by penetrating their surface can *a priori* not be denied.

Should one or another intraorganic migration route be followed in the definitive host, the infecting stage would evidently need the capacity to traverse host tissues. This is a feature worth emphasizing, because there are no other species of Brachylaimidae known to have acquired such a capacity to reach their final microhabitat. In Brachylaimidae, the adult stage of the great majority of species develops in the digestive tract (mainly intestine, rarely oesophagous and stomach). Only very few species present other microhabitats (*Scaphiostomum* in ducts of the liver and pancreas; *Dollfusinus* in nasal and frontal sinuses), but these are all directly connected to the digestive tract [14,16]. As already mentioned, the same applies to *Zeylanurotrema*, where both the reptilian and amphibian urinary bladders are part of the alimentary canal. In Brachylaimidae in general, it thus is the cercaria and not the metacercaria that presents a high tropism and migration capacity, a fact reflected by the existence of two postacetabular lateral aggregations of large penetration glands in cercariae. Nothing of this kind appears in the metacercariae [14,16,17]. This also supports a direct definitive-host-infection capacity by intrasporocystic cercariae and, thus, definitive host infection by predation of the first intermediate slug host in the case of *R. capensis*.

Owing to the adult stage microhabitats of kidneys and ureters, eggs are shed by the shrews by urinating. *A priori* this poses the problem of understanding how the first intermediate slug host may become infected by ingesting the fluke eggs dispersed throughout the external milieu. In other brachylaimids, eggs are in all cases shed with faeces and the fluke transmission is assured because of snails being attracted by the deposited stool materials, with many terrestrial gastropods showing coprophagous trends. Unfortunately, to our knowledge, nothing is known about potential snail attraction by urine. However, the shrew *M. varius* always urinate during the process of defaecation, therefore concomitantly depositing eggs with faecal material [69]. This fact has been corroborated by the finding of eggs of *R. capensis* on the shrew’s droppings.

## Conclusions

Characteristics of the morphology of the branched sporocysts, brevicaudate cercariae, unencysted metacercariae and life cycle pattern including two terrestrial slugs as first and second intermediate hosts, support the assignment of *R. capensis* to the family Brachylaimidae and subfamily Brachylaiminae, as previously proposed [39].

Epizootiological data, several morphological features, comparisons with other archaic and modern brachylaimids, and intraspecific mtDNA *cox*1 gene sequence variability suggest that *R. capensis* may use two transmission strategies in the study area of the Hottentots Holland Nature Reserve, South Africa. All indications are that *R. capensis* presents a very primitive condition, where the two-host life cycle is the normal modality, while the introduction of a second intermediate mollusc host is just in its early stages.

The kidney and ureter of the shrew, being the final microhabitat in *R. capensis*, is a unique feature in brachylaimids infecting mammals. This implies that the infecting stage must have the capacity to traverse the final host tissues in order to intra- and interorganically migrate from the digestive system to the urinary system. Such a requirement also supports a possible direct infection of the shrew by cercariae. Further support for this view is found in the observation that the shrew indeed does prey on the sporocyst and cercariae hosting slug *A. nebulosa*.

The three-host life cycle modality typical of brachylaimids thus seems to only be an additional, secondary and non-obligatory option for *R. capensis*, and even then only if its metacercariae, at least the young ones, still keep their capacity for such a complex tissue-traversing intraorganic migration. Consequently, in Brachylaimidae, the second intermediate mollusc host should evolutionarily be seen as a last addition to the life cycle. Furthermore, the present restriction of the adult stage to the microhabitat of the digestive tract and related organs of the final host must be regarded to result from a loss of the tissue-traversing capacity by the metacercarial stage.

## Competing interests

The authors declare that they have no competing interests.

## Authors’ contributions

WFS carried out the field collections and dissections of shrews and slugs, contributed the epizootiological studies and analyses, performed the first larval stage studies, and wrote an initial draft of the manuscript. PA carried out the DNA sequencing processes. MDB designed the sequencing study, analysed the sequences, and helped to draft the molecular part of the manuscript. SMC mounted and studied the larval stages, analysed the results, performed the literature review, made the drawings and photograph compositions, and wrote the final manuscript. All authors read and approved the final manuscript.
